# Precepts and practices in drug use indicators at government health care facilities: Hospital based prospective analysis

**DOI:** 10.4103/0253-7613.75682

**Published:** 2011-02

**Authors:** Hettihewa Lukshmy Menik, Amrasinghe I. Isuru, Subasinghe Sewwandi

**Affiliations:** 1 Department of Pharmacology, Faculty of Medicine University of Ruhuna, Sri Lanka,; 2 Molecular Science and Biomedical Unit, Faculty of Medicine University of Ruhuna, Sri Lanka; 3 Faculty of Medicine Pharmacy Unit, Allied Health Science Degree programme, Faculty of Medicine University of Ruhuna, Sri Lanka

Sir,

Good prescribing practice is an essential part of rational drug use.[1,2] A prescription audit therefore is a useful way of assessing the doctors’ contribution to rational use of drugs, and it can be evaluated with the world health organization (WHO) -recommended values.[3,4] In absence of adequate data on this subject in our country, we decided to study the degree of health care worker’s adherence to the principles of rational use of medicines in Galle, Sri Lanka.

This prescriber care assessment was carried out through an exit interview in 590 encounters of patients using a pretested, structured questionnaire. We used average consultation time (ACT), average number of drugs per encounter (ANDE), percentage of drugs prescribed by generic name (PDPG), percentage of encounters with an antibiotic prescribed (PAP), percentage of encounters with an injection prescribed (PIP) and percentage of drugs prescribed from essential drugs list or formulary (PEDL) to assess the degree of prescriber care and the situation in our health facilities.

Patient diagnosis and prescriber identity were absent in all prescriptions, although the signature was present in(nearly)all. Age was usually mentioned but sex was never mentioned in any of the 590 prescriptions. Duration of treatment and frequency of drug administration are usually mentioned. ACT, ANDE, PDPG, PAP and, PI, of three different hospitals are given in Table 1. Compared with the WHO (2008) -recommended figures, our ANDE was very high and PDPG was low. Use of generic names was significantly high. We found that use of antibiotics is much higher than the WHO-recommended values (20-26.8%) and use of injections was lower (13.4%-24.1%). Percentage of drugs prescribed from the essential drug list or formulary is compatible with the WHO values.

**Table 1:** Standard drug use indicators at different hospitals in Galle

*Prescribing and patient care indicators*	*Mean value (n = 590)*
Prescriptions without diagnosis	100%
Prescriptions without doctor’s identity	100%
Prescriptions without age of the patient	0.61%
Prescriptions without sex of the patient	100%
Prescriptions without treatment duration	0.61%
Prescriptions without treatment frequency	0.61%
Average consultation time (ACT) in min	1.77 ± 0.10
Average number of drugs per encounter (ANDE)	3.13
Percentage of encounters with an antibiotic prescribed (PAP)	58
Percentage of drugs prescribed by generic name (PDPG)	76
Percentage of encounters with an injection prescribed (PIP)	4.3
Percentage of drugs prescribed from essential drug list (PEDL)	98.6

We found that consultation time of a prescriber in government hospitals is very short as compared with the other reports.[1,2,5] The short ACT can be explained by the high patient:doctor ratio in our public sector health services. Although the diagnosis is an essential component in a prescription, it is never written. This is one of the common errors seen in government hospitals in our country and can be explained again with large number of patients being attended by a single medical officer. These issues should be addressed by the government to reduce the related complications. In contrast, frequency of drug administration and duration of the drug treatment was mentioned in more than 94% of the prescriptions. Significant adherence to use of essential drugs may be due to the relevant government policies, and is helpful to implement the cost-effective health strategies. Doctor’s identity remained absent in all prescriptions, which is serious as a prescriber may be needed in an emergency and hence they should be reiterated about the importance of patient doctor identity in a prescription. Low prescription of injections can be due to the large turnover of patients and establishment of emergency treatment units at the outpatient department. In contrast to that, prescription of antibiotics in our hospitals is very high, and this should be addressed as early as possible. Number of drugs per encounter is higher and can be related to unrealistic expectations from the patient, use of irrational drug combinations, unnecessary use of vitamins and aggressive promotion. We are happy to see that the maximum number of drugs from EDL have been used and it is probably because the procurement has to be as per The prescribed plans and policies.
